# Cure for the Tobacco Habit

**Published:** 1880-11

**Authors:** 


### CURE FOR THE TOBACCO HABIT.

Dr. F. R. Millard, in the *Medical and Surgical Reporter*, revives the question of the value of "pine-apple," as it is called, as a cure for the tobacco habit. This substance is a fungus that grows upon pine-trees. Dr. M. says that he has used it himself, and has prescribed it often, and has never seen a case in which it did not eventually eradicate the craving for tobacco. The taste is not unpleasantly bitter, and it is to be used about as a moderate chewer uses tobacco.

